# Lactate dehydrogenase (LDH) as an indicator of pre-eclampsia

**DOI:** 10.6026/973206300210116

**Published:** 2025-02-28

**Authors:** Kapil Raghuwanshi, Bhupesh Kushram, Dileep Dandotiya, Sudhakar Petkar, Swapnali Tambade, Mahendra Gandhe

**Affiliations:** 1 Department of Biochemistry, Chhindwara Institute of Medical Sciences, Chhindwara, Madhya Pradesh, India; 2 Department of Surgery, Chhindwara Institute of Medical Sciences, Chhindwara, Madhya Pradesh, India; 3 Department of Community Medicine, Chhindwara Institute of Medical Sciences, Chhindwara, Madhya Pradesh, India; 4 Department of Biochemistry, Chhindwara Institute of Medical Sciences, Chhindwara, Madhya Pradesh, India; 5 Department of Dentistry, Chhindwara Institute of Medical Sciences, Madhya Pradesh, India; 6 Department of Biochemistry, Chhindwara Institute of Medical Sciences, Chhindwara, Madhya Pradesh, India

**Keywords:** Preeclampsia, lactate dehydrogenase, placental hypoxia

## Abstract

High blood pressure (higher than 140/90 mm Hg), proteinuria and swelling due to fluid retention are symptoms of preeclampsia, a disease that affects pregnant women after the 20th week of pregnancy. The cytoplasm of cells undergoing anaerobic glycolysis contains the enzyme lactate dehydrogenase or LDH. Therefore, it is of interest to ascertain the blood lactate dehydrogenase levels of pre-eclamptic women, to assess and analyze these levels, to compare lactate dehydrogenase levels in different groups of preeclampsia patients and healthy controls and to examine the role of lactate dehydrogenase in preeclampsia severity ratings. Increased blood lactate dehydrogenase levels are associated with more severe preeclampsia, according to this study's results. Thus, it is crucial to determine lactate dehydrogenase levels in pre-eclamptic women early on so that these patients may get the right medicine and reduce the morbidity and mortality associated with these disorders.

## Background:

Proteinuria (*i.e.*, 300 mg or more in a 24-hour urine collection or 1+ on a dipstick) and hypertension (systolic blood pressure ≥140 mmHg and diastolic blood pressure ≥90 mmHg after 20 weeks of gestation) are the hallmarks of preeclampsia (PE). To identify severe PE, clinical criteria such as diastolic blood pressure of 110 mm Hg or higher, considerable proteinuria (dipstick measurement of 2+), or the presence of symptoms such as headache, diarrhea, convulsions, raised serum creatinine, thrombocytopenia, notable elevation of liver enzymes and pulmonary edema were examined [1]. Worldwide, preeclampsia affects 3- 10% of the population, with 8-10% of those affected living in India. A significant cause of maternal and perinatal mortality, PE affects 5-7% of pregnant women [2]. We still don't know what causes PE. Multiple studies have shown that preeclampsia is caused by a complex interplay of immune system issues, genetic predisposition, inflammation and malfunction of the maternal vascular endothelial cells, hypo perfusion of the placenta and abnormal invasion of trophoblasts. Because the extra-villous trophoblasts aren't very good at rebuilding the twisted uterine arteries, placental perfusion is low. First, in a normal pregnancy, the non-invasive trophoblastic shell develops during placentation. Then, there are two stages of trophoblastic invasion: step one: between weeks 10 and 12, the decidual portion of the spiral arterioles is invaded. Phase 2: Between 16 and 18 weeks of gestation, the myometrial segment of the spiral arterioles enters the intervillous area, dramatically boosting the flow of oxygenated maternal blood. Invasive trophoblastic growth is aided by a decrease in HIF-1 alpha expression, an increase in PO2 and the opening of intervillous space. The first stage of trophoblastic invasion in preeclampsia goes on as it should. However, the second stage, which involves penetrating the myometrial segment, doesn't happen due to defective trophoblastic differentiation. When the processes of trophoblastic invasion and vasodilatation do not succeed, placental hypo perfusion occurs. The rate of placental perfusion declines over the course of a pregnancy. Ischemia and hypoxia cause substances that harm and mal-function the endothelium to be discharged into the mother's circulation. Dysfunction or injury to the endothelium causes the following outcomes: Micro vascular coagulation and platelet aggregation result from increased capillary permeability, which allows fibrinogen and platelets to flow through the injured endothelium. In addition, it changes the ratio of prostaglandin production (thromboxane A2) to prostaglandin production (PGI2), which in turn causes the endothelium to secrete endothelin, which are vasoconstrictors. Endothelin, decreased NO and increased thromboxane A2 produce vasospasm in the small blood arteries of the end organs. The characteristics of preeclampsia are that the surrounding tissue haemorrhage becomes necrotic, which brings about the experience of ischemia. In microangiopathic hemolysis, factors such as platelet adhesion, fibrin deposition and endothelial damage all play a role. Consequently, elevated lactate dehydrogenase levels and schistocytes and spherocytes are seen in the peripheral smear [3]. Lactate is converted to pyruvic acid by the intracellular cytoplasmic enzyme lactate dehydrogenase (LDH), which results from anaerobic glycolysis. The normal range for serum lactate dehydrogenase levels in adults is 120-220 IU/L [4]. It is increased in preeclampsia due to prolonged hypoxia caused by placental ischemia and excessive anaerobic glycolysis and it is released into the circulation following cell death. Hypoxia in the placenta causes preeclampsia symptoms such as increased glycolysis and lactate dehydrogenase activity. Placentas from pre-eclamptic women are shown to have higher levels of gene expression and lactate dehydrogenase activity compared to those from healthy pregnant women, according to some studies [5, 6, 7]. Therefore, it is of interest to estimate the blood lactate dehydrogenase levels of pre-eclamptic women and compare lactate dehydrogenase levels in different groups of preeclampsia patients and healthy controls to examine the role of lactate dehydrogenase in preeclampsia severity ratings.

## Materials and Methods:

"Under the condition that they had obtained ethical approval, the case-control study was carried out by the departments of obstetrics and gynaecology and clinical biochemistry at CIMS Chhindwara M.P. One hundred and five pregnant ladies, ranging in age from eighteen to thirty-five, were scouted from the obstetrics and gynaecology department of CIMS Chhindwara M.P. Mild preeclampsia affected 35 pregnant and severe preeclampsia affected 35 pregnant women were taken as cases. For the control group, we used 35 identically aged pregnant women whose blood pressure was within the usual range. Every participant in the study gave their written informed consent.

## Inclusion criteria:

Each case happened in the third trimester of pregnancy and included a singleton, a woman's age being between 18 and 35, a woman's blood pressure being normal during the first 20 weeks of gestation and a woman's history of not having hypertension. (>28 weeks of gestation)"

## Exclusion criteria:

A chronic renal or hepatic problem, an infection chorioamnionitis an infection of the urinary system or a similar condition, combining drinking and smoking, medication for diabetes and having more than one fetus throughout a pregnancy. When the patient was fasting, a blood sample of 5 millilitres was obtained in a clot activator tube using aseptic procedures. To conduct biochemical experiments, serum was isolated and examined. Utilizing the turbidimetry technique on the Biosystem BA400, serum lactate dehydrogenase was measured. A completely automate analyzer for biochemistry purposes.

## Statistical analysis:

"The IBM SPSS program version 15 and a Microsoft Excel sheet were used to conduct quantitative and statistical studies. The data were presented as the mean, with a standard deviation of ±. The two groups' means were compared using the unpaired t-test. After running the data using one-way analysis of variance and post hoc Tukey, we compared the means of more than two groups. If the P-value was less than 0.05, we considered it statistically significant.

## Results and observations:

The demographic and biochemical characteristics of the people who participated in the research are shown in Table 1 & Table 2. Table 1 & Figure 1 (see PDF): The results of this study indicate that those diagnosed with severe preeclampsia had a considerably higher mean blood pressure of 161.20/116.86 mmHg. Patients who had moderate preeclampsia had a blood pressure reading of 138.97/90.06 mmHg. In contrast, pregnant women with normal blood pressure had a reading of 109.14/75.82 mmHg. For pregnant women who had severe preeclampsia, the serum uric acid level was 6.49 ± 0.87 mg/dl. On the other hand, for pregnant women who had moderate preeclampsia, the serum uric acid level was 4.65 ± 0.57 mg/dl. Finally, the serum uric acid level was 3.46 ± 0.45 mg/dl for pregnant women who had normotensive preeclampsia. The mean value of urine albumin in pregnant women who had severe preeclampsia was 1.07 ± 0.35 gm/day. In contrast, the mean value in pregnant women who had moderate preeclampsia was 0.62 ± 0.11 gm/day. In pregnant women who had normotensive preeclampsia, the mean value was 0.19 ± 0.05 gm/day. It was observed that there was a statistically significant difference in the mean blood pressure, serum uric acid and urine albumin level among pregnant women with severe preeclampsia, moderate preeclampsia and normotensive conditions (P< 0.00001 across the three groups)."

Unpaired 't' test applied. P value < 0.0001 was taken as statistically significant. Table 2 & Figure 2 (see PDF) a comparison and contrast of the case and control groups' serum lactate dehydrogenase levels is necessary. The average serum lactate dehydrogenase level in the control group was 218.60 ± 42.53 IU/L, but in the case group, it was 442.44 ± 74.05 IU/L. By comparing the case group to the control group, an examination of the data showed that the former had a significantly higher blood lactate dehydrogenase level (P < 0.0001).

Table 3 & Figure 3 (see PDF) Serum lactate dehydrogenase levels were 379.63 ± 47.55 IU/L in mild preeclampsia and 505.27 ± 27.28 IU/L in severe preeclampsia. In contrast, 218.60 ± 42.53 IU/L values were discovered in healthy pregnant women who were otherwise normal. A statistically significant difference was seen between the three groups of pregnant women with mild preeclampsia, severe preeclampsia and normotensive circumstances when the blood lactate dehydrogenase levels were analyzed (P< 0.00001). Table 4 shows a paired comparison of the study participants' blood lactate dehydrogenase levels. A statistically significant result (P < 0.00001) was obtained when comparing mild preeclampsia's mean serum lactate dehydrogenase levels with normotensive preeclampsia. Normotensive pregnant women had lower lactate dehydrogenase levels than those with mild preeclampsia, according to this study. A statistically significant result was obtained by comparing the mean serum lactate dehydrogenase levels between the two groups with normotensive and severe preeclampsia (P < 0.00001). The results showed that compared to pregnant women with normo-tension, those with severe preeclampsia had a greater level of LDH. Statistical significance (P < 0.00001) was found when comparing the mean serum lactate dehydrogenase levels between moderate and severe preeclampsia. A greater lactate dehydrogenase level was found in cases of severe preeclampsia as compared to cases of mild preeclampsia.

## Discussion:

Three to five per cent of pregnancies end in preeclampsia. Severe problems, including brain hemorrhage, renal failure and pulmonary edema, may also arise in preeclampsia. These consequences are linked to eclampsia, high liver enzymes and low platelet count (HELLP) syndrome. The placenta produces some pro-angiogenic (VEGF, PIGF) and anti-angiogenic (soluble fms-like tyrosine kinase-1, or sFlt1) and soluble endoglin (sEng) components. The production of pro-angiogenic and anti-angiogenic factors is balanced throughout a typical pregnancy. Anti-angiogenic factor production rises and pro-angiogenic factor production falls in PE due to placental hypo-perfusion and the ensuing hypoxia. This leads to endothelial damage and dysfunction and decreased vasodilator endothelium production (PGI2 & NO). One of the etiological factors contributing to preeclampsia is placental alterations. The myometrium's spiral arteries have a smaller diameter due to the failure of the second stage of trophoblastic invasion. Amorphous material replaces the cell wall when the vessel wall necrotizes. This leads to placental infractions and vascular obliteration. Preterm labour, placental abruption and fetal development limitation may result from these alterations. Increased syncytiotrophoblast degeneration, necrosis and apoptosis are seen. More syncytiotrophoblast micro-particles and debris are discharged into the mother's bloodstream, which causes endothelial dysfunction and inflammation. To detect endothelial dysfunction, placental hypo perfusion and other pathological alterations associated with preeclampsia, some assays have been developed. Most tests are not employed because they do not have sufficient sensitivity and predictive value.

The primary source of energy for placental cells is glycolysis. In preeclampsia, placental hypoxia leads to an upregulation of anaerobic glycolysis, increasing lactic acid synthesis by placental cells. Because of the lack of oxygen in the placenta, lactate dehydrogenase activity increases. There are five isoforms of LDH, with lactate dehydrogenase 4 being the one most affected by low oxygen levels in the placenta [8, 9]. The severity of preeclampsia and the extent to which placental cells are malfunctioning or damaged are indicated by elevated lactate dehydrogenase levels. Thus, monitoring the blood lactate dehydrogenase level in preeclampsia might be used as a biomarker to inform crucial treatment decisions, predict disease complications and ascertain the mother's and fetus's prognosis [10]. This study aimed to evaluate and compare the serum lactate dehydrogenase levels in pregnant women with preeclampsia with those of healthy pregnant women. Our study found that normotensive mothers had significantly lower serum lactate dehydrogenase levels than pre-eclamptic mothers (p-value < 0.0001) and that severe pre-eclamptic patients had significantly higher serum lactate dehydrogenase levels than mild pre-eclamptic and normotensive pregnancies (p-value < 0.00001). These findings are presented in Table 3 and Figure 3 (see PDF). These results were consistent with previous studies that indicated preeclampsia was associated with higher blood lactate dehydrogenase levels. According to Qublan *et al.* [11] the average levels of lactate dehydrogenase in healthy, normal pregnant women were 299 ± 79 IU/l. In patients with mild preeclampsia, the mean levels were 348 ± 76 IU/l, while in patients with severe preeclampsia, the mean levels were 774 ± 69.61 IU/l. A strong correlation was seen between serum lactate dehydrogenase levels and severe preeclampsia (P<0.001), as demonstrated by the findings. Jaiswar *et al.* [12] in their research found the levels of lactate dehydrogenase became significantly higher as the severity of the sickness rose (P<0.001), which is in an agreement with our findings. Research conducted by Sarkar *et al.* [13] revealed lactate dehydrogenase is a potential biochemical marker since it can be used to determine the severity of preeclampsia and may also have a role in the effective treatment of the condition. In contrast to the results of our research Nosrat *et al.* [14] discovered that there was no significant difference in serum lactate dehydrogenase levels between pre-eclamptic women and pregnant women in excellent health. The current research was developed to determine the blood lactate dehydrogenase level in pregnant women who were diagnosed with moderate or severe preeclampsia.

## Conclusion:

Pregnant women who are at risk of getting preeclampsia must be identified as soon as possible because they need to be closely monitored and treated appropriately to improve the quality of their pregnancy. Clinical criteria, which are based on clinical presentation, are often used to diagnose pre-eclampsia. Currently, there is no clinically accepted standard diagnostic test. We show that a higher blood lactate dehydrogenase level is linked to the severity of preeclampsia. They may be used as a prognostic indicator starting in the first trimester. All pregnant women may thus have their blood lactate dehydrogenase levels evaluated to predict preeclampsia and high-risk pregnant women may benefit from routine serum lactate dehydrogenase level monitoring for early diagnosis and management to reduce maternal and fetal morbidity and death.

## Funding:

Nil

## Figures and Tables

**Table 1:** Comparison of demographic & clinical profile between cases and controls

**Variables**	**Normotensive Pregnant women (n=35)**	**Pregnant Women with Mild Preeclampsia PE (n=35)**	**Pregnant Women with Severe Preeclampsia (n=35)**	**F-Value**	**P-Value**
Age (years)	24.37± 1.40	24.20± 4.26	23.17± 1.74	1.9113	0.1531
Systolic Blood Pressure(mmHg)	109.14± 7.08	138.97± 5.41	161.20± 7.31	539.032	P < 0.00001
Diastolic Blood Pressure(mmHg)	75.82± 5.95	90.06± 6.09	116.86± 5.72	433.139	P < 0.00001
Serum Uric acid (mg/dl)	3.46± 0.45	4.65± 0.57	6.49± 0.87	189.075	P < 0.00001
Urine albumin (gm/day)	0.19± 0.05	0.62± 0.11	1.07± 0.35	150.461	P < 0.00001

**Table 2:** Comparison of Serum lactate dehydrogenase between the case and control groups

**Parameter**	**Case group (n=70) (Mean± SD)**	**Control group (n=35) (Mean± SD)**	**T value**	**P value**
Serum lactate dehydrogenase (IU/L)	442.44± 74.05	218.60± 42.53	16.5452	< 0.0001

**Table 3:** Comparison of mean serum lactate dehydrogenase in study subjects

**Pregnant Women**	**N**	**Mean lactate dehydrogenase (IU/L)**	**Std. Deviation**	**F Test**	**P Value**	**Result**
Normotensive	35	218.6	42.53	450.4118	< 0.00001	Significant
Mild Preeclampsia	35	379.63	47.55			
Severe Preeclampsia	35	505.27	27.28			
One-way ANOVA applied. P value < 0 .00001, Significant

**Table 4:** Pair-wise comparison of serum lactate dehydrogenase by post-hoc tukey test

**Pair**	**F factor**	**P value**	**Interpretation**
Normotensive-Mild Preeclampsia	223.00884	<.00001	Significant
Normotensive-Severe Preeclampsia	1126.78066	<.00001	Significant
Mild Preeclampsia-Severe Preeclampsia	183.8455	<.00001	Significant
